# 168. Promising Changes In Antibiotic Utilization Associated With Implementation Of Rapid Multiplex Polymerase Chain Reaction Testing For Bloodstream Infection

**DOI:** 10.1093/ofid/ofad500.241

**Published:** 2023-11-27

**Authors:** Tat Yau, Michelle Blyth, Julio E Figueroa

**Affiliations:** LSU Health Sciences Center New Orleans, New Orleans, Louisiana; LSU Health Sciences Center-New Orle, New Orleans, Louisiana; Louisiana State University Health, School of Medicine, New Orleans, Louisiana, New Orleans, LA

## Abstract

**Background:**

BioFire® Blood Culture Identification 2 (BCID2) panel is a rapid multiplex polymerase chain reaction (rm-PCR) testing for 43 organisms associated with bloodstream infections and 10 antimicrobial resistance genes. Previous literature suggested combination of rm-PCR and antibiotic stewardship program (ASP) would improve time to organism identification, optimal therapy, and de-escalation of antibiotics.

**Methods:**

This study was a retrospective study at Touro Infirmary in New Orleans, Louisiana. All adult patients with positive blood cultures before and after BCID2 implementation in January 2022 (April to September 2021 and April to September 2022 respectively) were included. The outcomes of interest included time to organism identification, time to change of antibiotics (either appropriate escalation or de-escalation), hospital length of stay, 30-day re-admission, and 30-day mortality.

**Results:**

A total of 117 patients met the study criteria (refer to Figure 1 and Table 1 for the flowchart of participants and characteristics of participants respectively). Patients with blood cultures that grew one of the organisms of interest were analyzed: methicillin sensitive Staphylococcus aureus (MSSA), coagulase negative Staphylococcus species, extended-spectrum beta-lactamase-producing Enterobacterales (ESBL-E), AmpC beta-lactamase-producing Enterobacterales, and Pseudomonas aeruginosa. There was a significant difference in the time to organism identification between pre- and post-BCID groups (45 hours vs. 25.1 hours, p < 0.0001) and there was no discordance between the BCID and blood culture results. In addition, there was a significant decrease in time to antibiotics change between pre- and post-BCID groups (p < 0.01). Notably for patients with MSSA bacteremia, post-BCID group had significantly shorter duration to de-escalate antibiotics than pre-BCID group (43 hours vs. 66.7 hours, p < 0.038). However, there is no statistical difference between pre- and post-BCID groups regarding hospital length of stay, 30-day readmission, and 30-day mortality.

Flowchart of Participants

**Figure:**
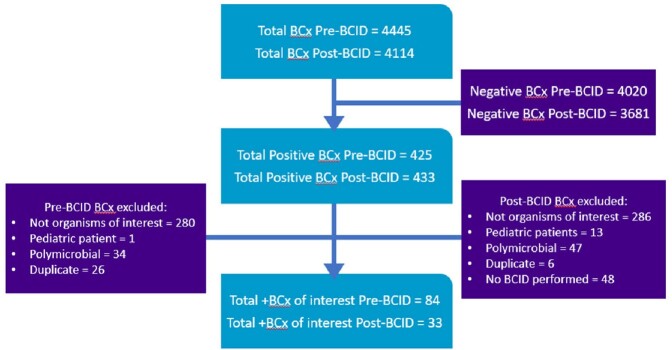

Organisms of interest include: methicillin sensitive Staphylococcus aureus, coagulase negative Staphylococcus species, extended-spectrum beta-lactamase producing Enterobacterales, AmpC beta-lactamase producing Enterobacterales, and Pseudomonas aeruginosa. Abbreviations: BCID, blood culture identification panel, BCx, blood cultures.

Characteristics of Participants

**Figure:**
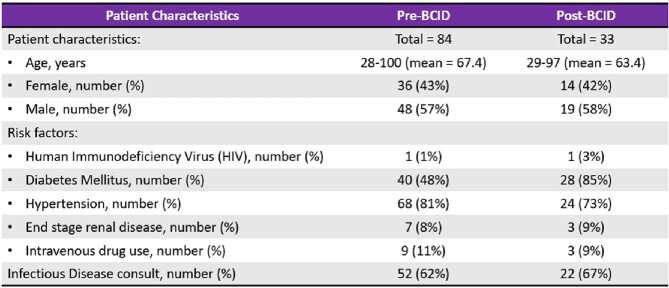

**Conclusion:**

BCID2 significantly reduced the time to organism identification and improved time to effective therapy for MSSA bacteremia. However, significance was limited in other measures due to small sample size.

**Disclosures:**

**All Authors**: No reported disclosures

